# An Essential Role for Diet in Exercise-Mediated Protection against Dyslipidemia, Inflammation and Atherosclerosis in ApoE-/- Mice

**DOI:** 10.1371/journal.pone.0017263

**Published:** 2011-02-16

**Authors:** Liliana Cesar, Samuel Vasallo Suarez, Jennipher Adi, Nikhil Adi, Roberto Vazquez-Padron, Hong Yu, Qi Ma, Pascal J. Goldschmidt-Clermont, Arthur Agatston, Paul Kurlansky, Keith A. Webster

**Affiliations:** 1 Department of Molecular and Cellular Pharmacology and the Vascular Biology Institute, University of Miami Miller School of Medicine, Miami, Florida, United States of America; 2 Florida Heart Research Institute, Miami Beach, Florida, United States of America; 3 Agatston Research Institute, Miami Beach, Florida, United States of America; University of Padova Medical School, Italy

## Abstract

**Background:**

Diet and exercise promote cardiovascular health but their relative contributions to atherosclerosis are not fully known. The transition from a sedentary to active lifestyle requires increased caloric intake to achieve energy balance. Using atherosclerosis-prone ApoE-null mice we sought to determine whether the benefits of exercise for arterial disease are dependent on the food source of the additional calories.

**Methods and Results:**

Mice were fed a high-fat diet (HF) for 4.5 months to initiate atherosclerosis after which time half were continued on HF while the other half were switched to a high protein/fish oil diet (HP). Half of each group underwent voluntary running. Food intake, running distance, body weight, lipids, inflammation markers, and atherosclerotic plaque were quantified. Two-way ANOVA tests were used to assess differences and interactions between groups. Exercised mice ran approximately 6-km per day with no difference between groups. Both groups increased food intake during exercise and there was a significant main effect of exercise F((1,44) = 9.86, p<0.01) without interaction. Diet or exercise produced significant independent effects on body weight (diet: F(1,52) = 6.85, p = 0.012; exercise: F(1,52) = 9.52, p<0.01) with no significant interaction. The combination of HP diet and exercise produced a greater decrease in total cholesterol (F(1, 46) = 7.9, p<0.01) and LDL (F(1, 46) = 7.33, p<0.01) with a large effect on the size of the interaction. HP diet and exercise independently reduced TGL and VLDL (p<0.05 and 0.001 respectively). Interleukin 6 and C-reactive protein were highest in the HF-sedentary group and were significantly reduced by exercise only in this group. Plaque accumulation in the aortic arch, a marker of cardiovascular events was reduced by the HP diet and the effect was significantly potentiated by exercise only in this group resulting in significant plaque regression (F1, 49 = 4.77, p<0.05).

**Conclusion:**

In this model exercise is beneficial to combat dyslipidemia and protect from atherosclerosis only when combined with diet.

## Introduction

Lifestyle modifications including diet, exercise, and weight control are recommended for the treatment of dyslipidemia and associated coronary artery disease (CAD). The benefits of each of these modifications on health are dose-responsive. ACSM/CDC/AHA guidelines recommend a minimum of 30 minutes of moderate-intensity physical activity per day with an open-ended maximum [1]. Dietary recommendations include caloric intake appropriate for maintenance or reduction of body weight, reduced consumption of sugars, saturated fats and processed foods, and replacement with protein, unsaturated fats, omega-3 and complex carbohydrates. A body mass index (BMI)<25 kg/m2 is recommended for optimal cardiovascular health.

Serum lipids are strong mediators of CAD and indicators of cardiovascular risk. Atherogenic dyslipidemia is characterized by abnormally low serum concentrations of HDL cholesterol and elevations of triglycerides (TG), low-density (LDL) and very low-density (VLDL) lipoprotein-cholesterol. Much of the reduction in cardiovascular morbidity and mortality in Western societies over the past 2 decades has been attributed to the benefits of more effective control of serum lipids through lifestyle changes and pharmacological management [2]–[7]. Epidemiological studies have shown that exercise training without dietary intervention leads to only minimal reductions of body mass but, as expected this is markedly improved by concomitant diet modulation [reviewed in [7]–[15]]. The individual roles of diet, exercise and body mass in lipid regulation and CAD are complex because these parameters do not act independently [16]–[18]. Even after separation into weight categories and correction for dietary intervention there are marked inconsistencies between, and sometimes within studies on the effect of exercise on plasma lipids. A meta-analysis of 61 study groups and 2200 subjects showed that endurance exercise training alone lead to significant reductions of TG, LDL and total cholesterol (TC) in less than 50% of cases [19]. These numbers were potentiated by concomitant dietary intervention in most studies but dietary fat reduction also tended to reduce HDL. These studies indicate that in human subjects it is still not possible to predict the effects of exercise without diet modification on atherogenic lipid profiles or associated CAD.

While the influence of exercise alone on blood lipids is unclear, definitive studies have confirmed positive effects of both moderate and high-intensity exercise training on molecular parameters that determine the so-called metabolic syndrome [20]. Exercise has been shown to increase production of nitric oxide, reduce systemic inflammation and increase levels of circulating endothelial progenitor cells [21]–[26]. Exercise training is now an established therapeutic intervention with benefits that include enhancement of myocardial and peripheral perfusion and reduction of morbidity and mortality of patients with CAD [reviewed in [27], [28]]. In a prospective clinical study of CAD patients, 4-weeks of intensive exercise training decreased acetyl-choline-induced coronary artery vasoconstriction by 54%, an effect that was sustained with continued exercise [29], [30].

The effect of combined exercise and diet on lipid profile and atherosclerosis is still an open question. Studies on ApoE-/- mice demonstrated favorable effects of treadmill running or swimming on plaque reduction after carotid injury or hypercholesterolemia respectively [31]–[33]. In both cases short exercise periods reduced inflammatory markers and decreased plaque. The effects were deemed to be independent of systemic lipids and were attributed to anti-oxidant and anti-inflammatory effects. Here we tested the effects of radical lifestyle modifications including voluntary running (6-km/day) and ad-lib high fat (HF) or high protein/fish oil (HP) diets on ApoE -/- mice with pre-developed plaque. The results show that exercise positively modifies lipid profiles and atherosclerotic plaque accumulation only when combined with the HP. Diet and lipid profiles correlated closely with atherosclerosis. Inflammatory markers IL6 and CRP were both increased by feeding HF chow and this was blunted by exercise.

## Materials and Methods

### Animals

Male ApoE-/- mice 5 weeks of age were purchased from Jackson laboratories (Bar Harbor, Maine) and handled according to University of Miami animal care and use regulations. Mice were kept in rooms with alternating 12-hour periods of light and dark with ad-libitum access to water and chow. For the first 4.0 months after arriving all mice were fed high fat chow diet #88137 (Harlan-Teklad; 42% fat, 1.25% cholesterol; (HF)) beginning at 5 weeks of age. After 4.0 months, 6 animals were sacrificed as a baseline for aortic atherosclerotic plaque, the other mice were individually housed in cages either equipped with computer monitored running wheels (exercise group; n = 20) or not (non-exercise group; n = 20). Half of each group were continued on the HF diet and the other half were switched to a diet high in protein and unsaturated oils (custom diet from Purina; 45% protein, 39% fat (Canola and Menhaden fish oil (1∶1); (HP)) also at 4.0 months. For diet switching, mice were weaned off the HF diet by gradually increasing the ratio of HP: HF chow over 2 weeks. Mice were sacrificed and aortas harvested after a further 2.5 months so that the overall duration of the study was 6.5 months and mice were aged 7.75 months at the time of sacrifice. The HP chow was created by replacing all animal fat from the HF formula with Canola and fish oils while keeping total calories from fat at approximately 40%. Calories from protein were also increased (to 45%) at the expense of carbohydrates (to 15%) in the HP diet. Distance and time of running were recorded continuously, body weights were determined weekly and average weekly food intakes were monitored.

### Blood plasma lipids and cytokines

Blood samples were taken monthly from the orbital sinus after gas anesthesia, plasma was separated and stored at −80°C until analysis. Total cholesterol, HDL, triglycerides, LDL and VLDL were measured by reflectance spectrophotometry (VITROS Chemistry System Ortho-Clinical Diagnostics, Raritan, NJ) using the manufacturer's reagents and protocols. C-Reactive Protein (CRP) was measured by Rat/Mouse CVD CRP Single Plex (Millipore Corp. Bedford, MA) and IL-l alpha and β, IL-6, IL-10, IFN-gamma, and TNF-alpha, by Milliplex Mouse Cytokine Panel 6-plex (Millipore Corp. Bedford, MA). Mean values represent data taken during the full time course after implementation of the exercise regimens.

### Atherosclerosis

Whole aortas were opened lengthwise, fixed in 10% formalin, stained with oil red O and quantified by computerized morphometrics. The results for atherosclerotic plaque were expressed as the mean percent of baseline values and represent the mean ± standard error of the mean.

### Statistical analysis

Statistical analysis was carried out using the Statistical Package for the Social Sciences (SPSS, Inc., Chicago, IL, USA). All data are expressed as means ± SEM unless noted otherwise. The results for atherosclerotic plaque are expressed as the mean ± SEM percent of baseline values. A level of 5% probability was considered significant. The normality of distribution of each variable was tested and transformed data were used when necessary. Differences between two groups were analyzed by Student's t test and Mann-Whitney U test. A two-way between groups ANOVA was used to evaluate diet and exercise interaction effects for dependent variables; a significant interaction was interpreted by a subsequent simple-effects analysis with Bonferroni correction.

## Results

### Exercise rates, food intake and body weight

**Figure 1:**
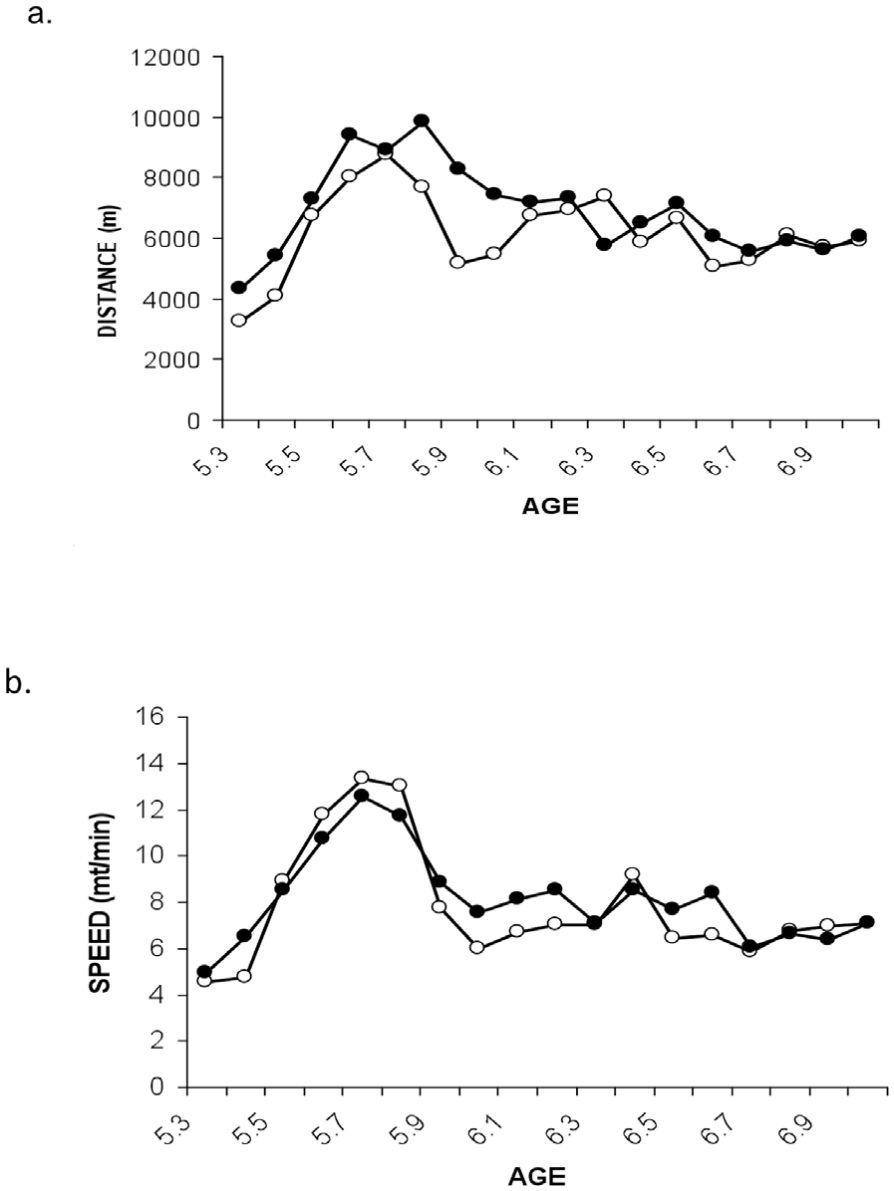
Exercise parameters of mice fed HF and HP diets. Mice were received at age 5-weeks and fed HF for 4.5 months as described in Methods. At this time half of the mice were randomly assigned to continue on the HF diet and half were weaned off the HF diet onto the HP diet as described in Methods. At the same time half of the mice from each diet (10 mice per group) were randomly assigned to exercise by housing in individual cages with computer-monitored exercise wheels or remained sedentary also in individual cages without wheels. Running was monitored continuously. Each point is the mean of 10 mice per group; exercised mice only. After a lag period and slight overshoot, mean running distance stabilized at 6-km per day. Open circles HP; closed circles HF.

**Figure 2:**
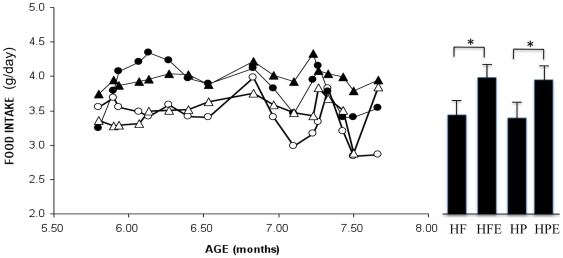
Comparison of food intake between groups. Food consumption was measured 3 times per week in all groups by weighing chow pellets and calculating mean consumption for each group (n = 10). Open circles: high protein (HP) sedentary; closed circles: HP-exercise; open triangles: high fat (HF) sedentary; closed triangles: HF exercise. Bars on right are mean of total food intake during the exercise period ± SEM; both exercise groups consumed significantly more chow than the sedentary groups; p<0.01 by Mann-Whitney U test.

**Figure 3:**
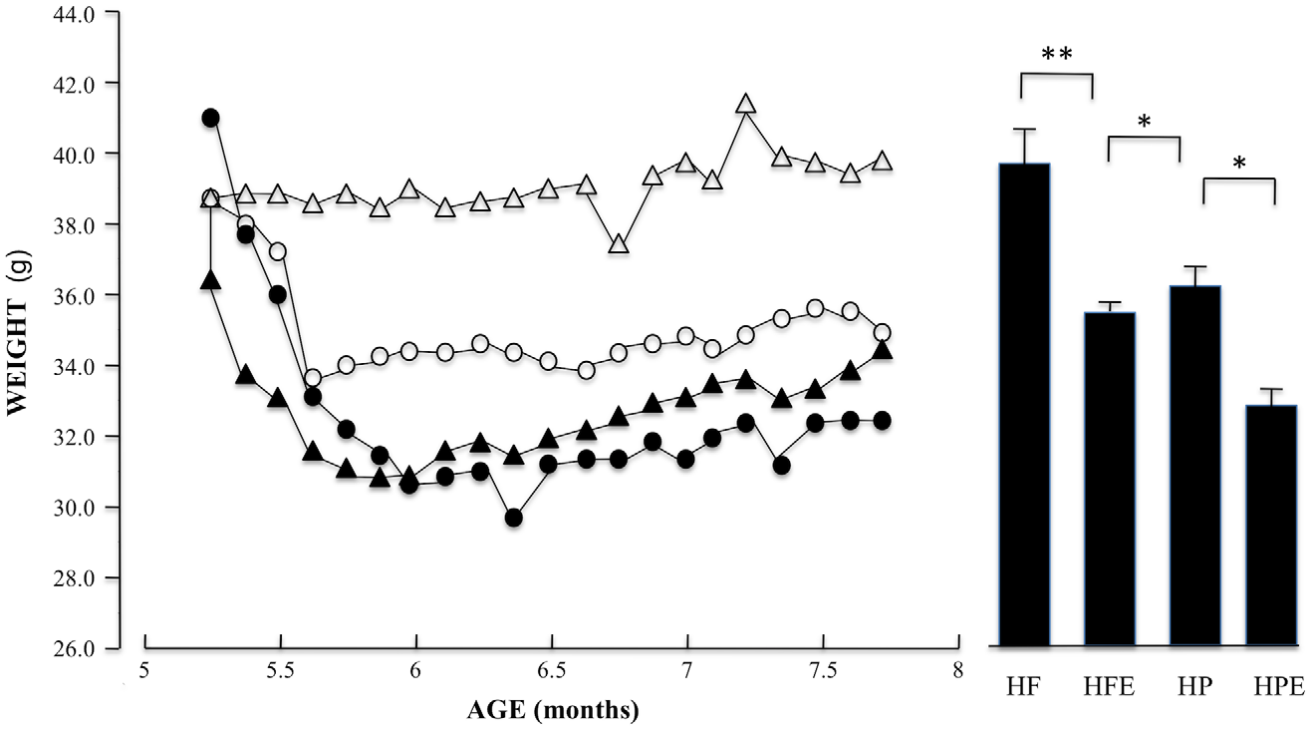
Comparisons of total body weight between groups. Mice were weighed 3 times per week in all groups and mean weight calculated (n = 10 per group). Symbols as in Figure 2. For bar graphs on the right each value represents the mean of body weights during the exercise period ± SEM; data was analyzed by two-way ANOVA as described in Methods; analysis of simple effects; **p<0.001; *p<0.01).

Mean values for speed of running and distance are shown in Figure 1. Distance increased to almost 10 km per day over the first 2 weeks and then dropped and stabilized at about 6 km. There was a small non-significant trend for the HF diet group to out-run the HP group (HF 6.88±0.35 km/day; HP 6.15±3.2 km/day; p = 0.13). Average speed followed similar trends again with no significant difference between groups (p = 0.23). As shown in Figure 2, food intake was increased in both diet groups during the first 4 weeks of exercise and stabilized with significantly more food intake by the exercise relative to the sedentary groups. This was confirmed by two-way ANOVA between groups analysis that showed a significant main effect for exercise F (1,44) = 9.86, p<0.01) without diet/exercise interaction. The diet main effect was not significant indicating that animals consumed the same amount of food on both diets. Body weights fell during the first 6 weeks of diet and exercise and stabilized in these groups thereafter but continued to increase in HF sedentary group (Fig. 3). Two-way ANOVA revealed no significant interaction between diet and exercise on body weight but very significant main effects for diet (F (1,52) = 6.85, p<0.01) and exercise (F (1,52) = 9.5, p = 0.01), indicating that these factors acted independently. The switch from HF to HP resulted in a significantly reduced mean body weight over 3 months (38.7±1.4 g vs. 36.2±0.7 g, p<0.01 for HF and HP groups respectively). Similarly, exercising mice weighed significantly less than their sedentary counterparts (HF sedentary, 38.7±1.4 g vs. HF-E 35.1±0.2 g, p<0.01). Exercising mice fed HP had the lowest mean body weight that was significantly lower than the sedentary HP group (HP, 36.2±0.7 g vs. HPE, 33.1±0.5 g). It is noteworthy that mean weight of mice in the HF-E group was less than that of sedentary mice in the HP group (HF-E, 35.1±0.2 g vs. HP, 36.2±0.7 g) suggesting that exercise is superior to diet in preventing weight gain in these mice. It is also noteworthy that exercise resulted in reduced body weight in both diet groups despite significantly increased food intake relative to the sedentary groups.

### Lipid profiles

**Figure 4:**
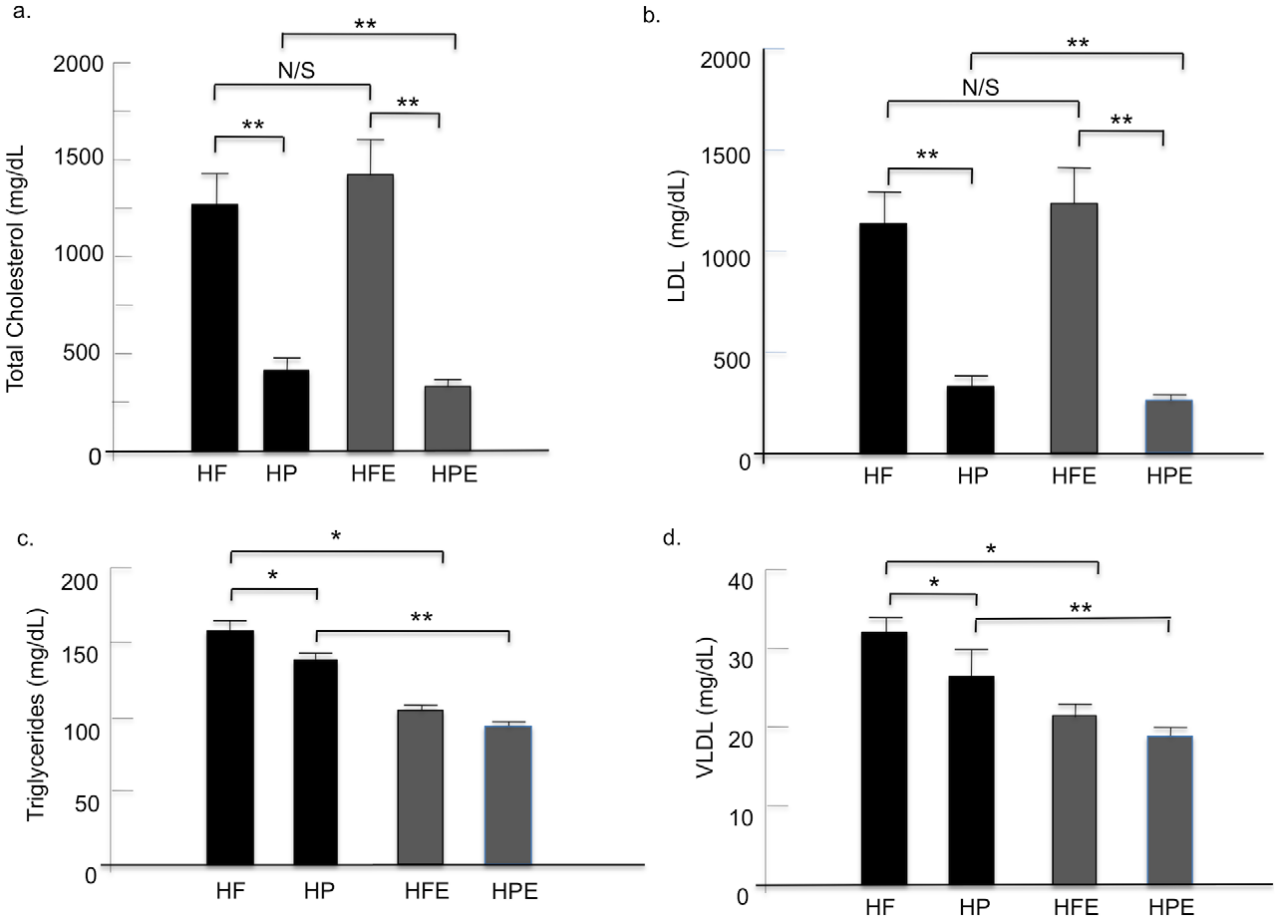
Comparisons of lipid profiles between groups. Blood was collected at the time of sacrifice in all groups. Total cholesterol (TC), HDL, triglycerides (TG), LDL and VLDL were measured by gas chromatography. HF, HP, HFE, HPE as in Figures 1 and 2. Each value represents the mean plasma lipid level for the exercise period ± SEM; two-way ANOVA was used as described for Figure 3; **p<0.001; *p<0.05.

**Figure 5:**
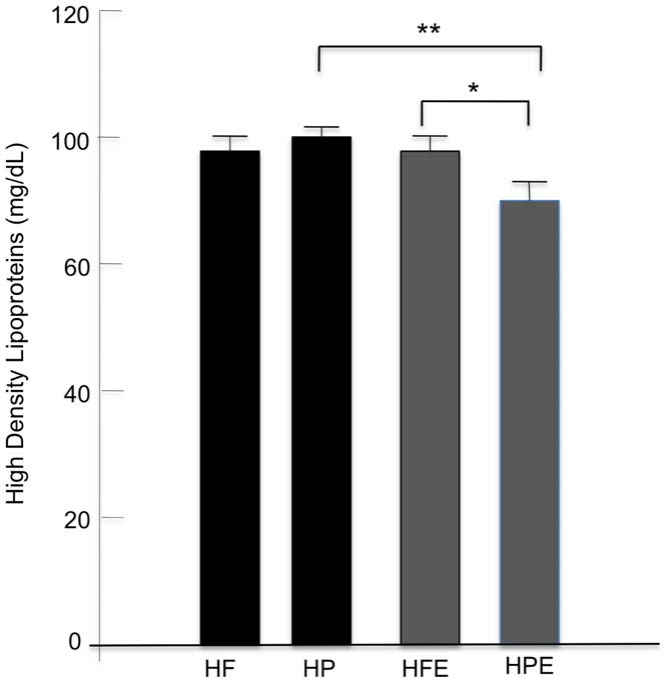
HDL profiles. HDL was measured in blood samples by gas chromatography as described for Figure 4; labeling and statistics also as described in Figures 3 and 4; *p<0.05; **p<0.001.

Changes in plasma lipids are shown in Figures 4 (a–d) and 5. Total cholesterol and LDL sustained the most dramatic changes (Figs. 4 a & b). A positive interactive effect of diet and exercise on TC (F (1, 46) = 7.9, p<0.01) and LDL (F (1, 46) = 7.33, p<0.01) was confirmed by 2-way ANOVA. Main effects for diet and activity status were also significant. The switch from HF to HP resulted in marked declines of both TC and LDL with >2-fold decrease of TC (p<0.001) and almost 3-fold decrease of LDL (p<0.001) in the HP group (HF-TC, 1267±178; HP-TC, 455±77 mg/dL; HF-LDL, 1138±178; HP-LDL, 329±72 mg/dL). Further analysis of the interactive effect indicated that exercise failed to decrease either lipoprotein levels in animals that were continued on the HF diet (HF-TC, 1267±178; HFE-TC, 1365±196 mg/dL, p = 0.71; HF-LDL, 1138±178; HFE-LDL, 1246±196 mg/dL, p = 0.69); however exercised mice in the HP group displayed further significant decreases of both TC and LDL (HP-TC, 455±77 mg/dL; HPE-TC, 360±25 mg/dL, p = 0.001; HP-LDL, 329±72 mg/dL; HPE-LDL, 263±19.2 mg/dL, p = 0.001). Triglyceride (TG) and VLDL levels were lower in the HP groups compared to HF animals (p<0.05); and were significantly decreased by exercise in both groups (p<0.001). Two-way ANOVA did not reveal a significant interaction between diet and exercise on TG or VLDL. Instead, main effects were confirmed for diet (TG, F (1,46) = 6.1, p = 0.017; VLDL, F (1,46) = 6.4, p = 0.015) and exercise (TG, F (1,46) = 41.1, p<0.001; VLDL, F (1,46) = 40.4, p<0.001). The added main effects of diet and exercise resulted in the lowest plasma levels of TG and VLDL for animals in the HP-E relative to HF-E groups (TG, 95±5.2 vs. 105±3.6 and VLDL, 19±1.0 vs. 21±1 mg/dL). HDL was not significantly affected by diet alone, (Figure 5) but the interaction of diet with exercise (F (1,46) = 4.2, p<0.05) caused a marked drop of HDL in the HP-E group (HP, 100±3.4 mg/dL; HP-E 78±6 mg/dL, p<0.05). The results demonstrate independent effects of diet and exercise on TG and VLDL but a strong interactive effect on TC and LDL; a combination of HP diet and exercise was required to lower TC and LDL, two of the strongest metabolic risk factors for coronary artery disease. Importantly, we found that HDL levels were significantly lower in the HP-E group. The reasons for this are not clear however the HP-E group had the lowest body weight, significantly lower than the HP sedentary group (Figure 3), combined with low dietary cholesterol and saturated/trans fat. Previous studies on human subjects that have reported decreased HDL levels associated with diets that are low in saturated fat [19], [34], [35].

### Cytokines

**Figure 6:**
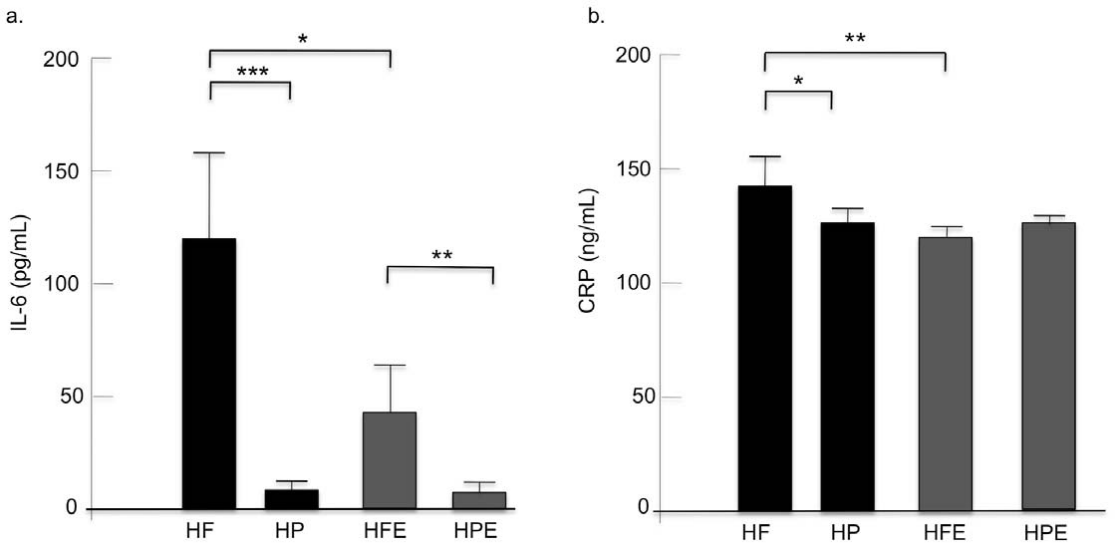
Comparison of inflammation markers between groups. Blood was collected as described in the Figure 4 legend. Interleukin-6 (IL-6) was quantified in serum samples by ELISA using a Milliplex Mouse Cytokine Panel 6-plex as described in Methods. C-reactive protein (CRP) was quantified in plasma by using a CVD CRP Single Plex kit also described in Methods. In data not shown we observed no changes in IL-1α and β, IL-10, IFN-γ, and TNF-α, by the same assays. Statistical analysis are as described for Figure 3; ***p<0.001; ** p<0.01; * p<0.05.

Figure 6 shows changes in serum inflammatory markers associated with diet and exercise groups. Results for IL6 and CRP followed similar trends. IL6 levels were markedly decreased in animals fed the HP relative to HF diet (HP, Md = 6.4 pg/ml; HF, Md = 61.1 pg/ml, p<0.01). Likewise for CRP, switching to the HP diet significantly reduced plasma levels (HP, 127.4 ng/ml; HF, 135 ng/ml, p<0.05). In the HP diet group exercise did not significantly affect the plasma levels of either IL6 or CRP (HP-IL6, Md = 6.4 pg/ml; HPE-IL6, Md = 6.4 pg/ml, p>0.05; HP-CRP, 127.4 ng/ml; HPE-CRP, 128.0 ng/ml, p>0.05) meaning that exercise did not further augment the already powerful effect of HP diet alone. Contrary to this, exercise caused a significant 8-fold reduction of IL6 and CRP in the HF diet group, (HF-IL6, Md = 61.1 pg/ml; HFE-IL6, Md = 8.1 pg/ml, p<0.05; HF-CRP, 135.0 ng/ml; HFE-CRP, 122.0 ng/ml p<0.01). In this case 2-way ANOVA revealed a trend for positive interaction between diet and exercise on CRP (p = 0.052). These results are also consistent with the possibility that CRP is regulated by IL-6 [36]. We observed no significant changes in the levels of TNF-alpha, IL10, IFN-gamma or IL1β (not shown).

### Atherosclerosis

**Figure 7:**
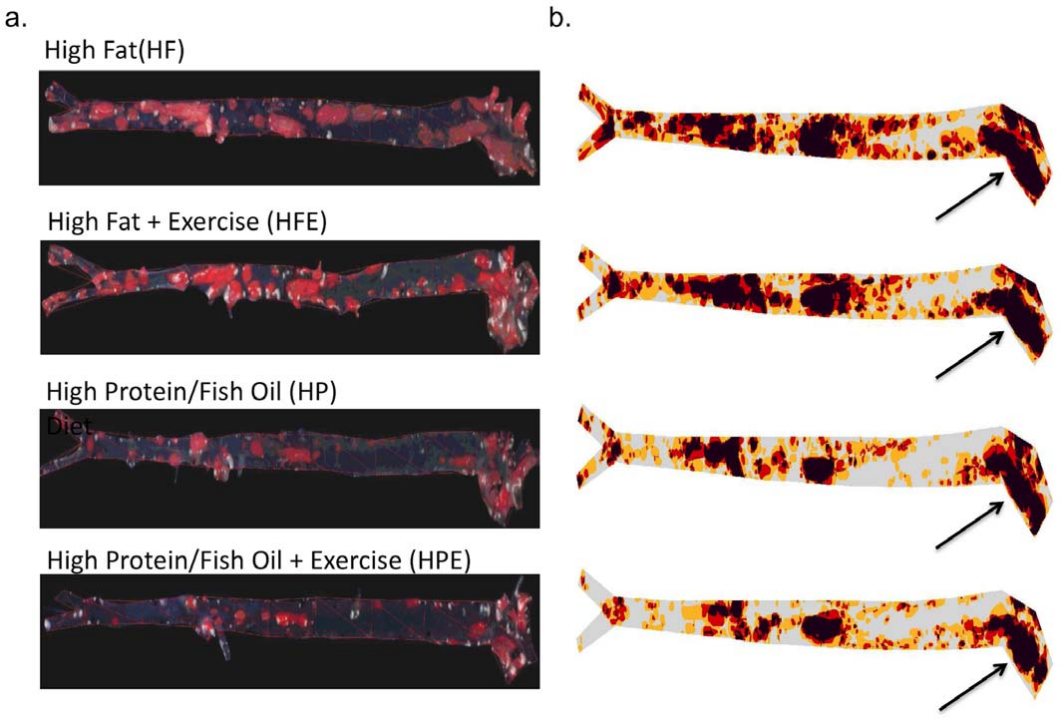
Comparisons of atherosclerotic plaque accumulation between groups. Aortas were harvested at the time of sacrifice (8-months), fixed in paraformaldehyde and stained with Oil-Red as described in Methods. Left panels show light microscope view of aortas representative of each condition. Right panels show subtraction images used for plaque quantification by densitometry (the method is described in detail in Ref 49).

**Figure 8:**
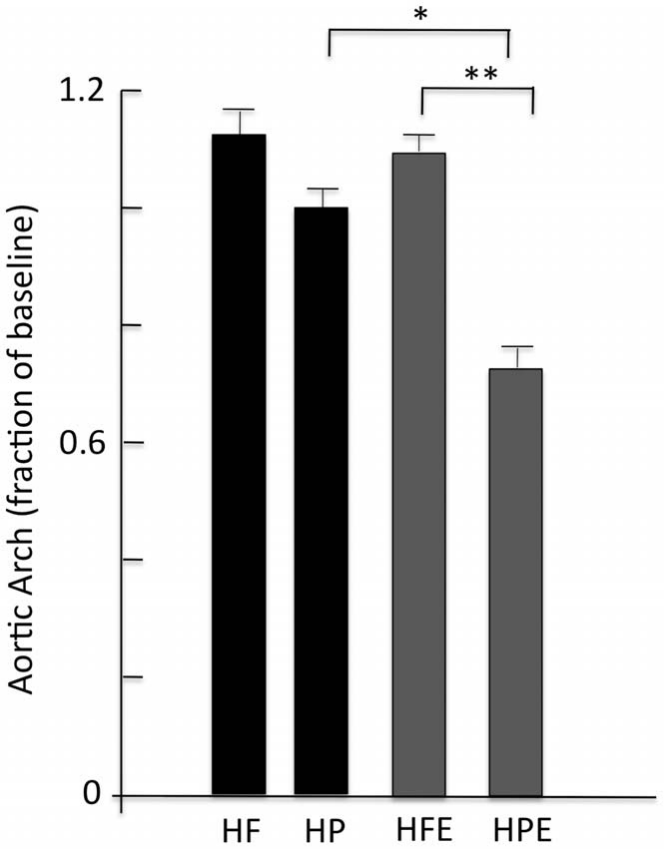
Quantification of aortic arch plaque accumulation in different groups. Aortas were harvested and processed as described in Figure 6 legend and Methods. Plaque in the arches of 8–10 aortas from each group was quantified as described in Methods and expressed as percentages of the baseline plaque at 5-months. Statistics are by 2-way ANOVA as described for Figure 3; *p<0.05; ** p<0.01.

Figure 7 shows representative examples of aortas from mice treated as indicated and stained en-face with Oil-Red as described in Methods. Arrows indicate the aortic arch where plaque accumulation in effected subjects is usually the highest and is a strong predictor for cardiovascular events including stroke [37]. Figure 8 shows the quantification of aortic arch plaque in the four experimental groups expressed as the mean ± SEM percent of baseline values. Decreased plaque levels associated with HP ± diet are apparent in the aortas, and two-way ANOVA analysis confirmed that modification of the diet only did not account for a reduction in plaque (HF, 1.12±0.06; HP, 1.01±0.05, p = 0.18). Similarly, exercise did not reduce plaque accumulation in the HF group relative to the sedentary counterparts (HF, 1.12±0.06; HFE, 1.06±0.04, p = 0.42). This is consistent with the effects of exercise on TC and LDL in this group and may reflect the increased intake of saturated fat that was associated with exercise. Two-way ANOVA revealed a significant interaction between diet and exercise in plaque reduction (F (1, 49) = 4.77, p<0.05) in the aortic arch and main effects for diet (p<0.001) and activity status (p<0.01). Therefore both diet and exercise contribute to plaque reduction, and the combination conferred significant protection relative to all other groups (HP, 1.01±0.05; HP-E, 0.71±0.05; F (1,49) = 15.4, p<0.001). These changes are consistent with parallel combined effects of the HP diet and exercise on TC and LDL.

## Discussion

Our results show for the first time that exercise without dietary intervention did not favorably benefit atherogenic lipids (TC, LDL) or plaque accumulation on the aortic arch in ApoE knockout mice with advanced atherosclerosis. This was despite significant exercise-mediated reduction of inflammatory markers IL-6 and CRP in the HF group. A similar effect of exercise on pro-inflammatory cytokine reduction was observed in a prior study by our group also using the ApoE knockout mice model [38]. Conversely, exercise combined with a diet enriched in protein and unsaturated oils conferred optimal protection against atherosclerosis, with significantly improved inflammatory markers, lipid profiles, and reduced plaque accumulation. TC, LDL, and IL6 each decreased by >2-fold when mice were switched from HF to HP, and exercise resulted in an additional 25% decrease of both lipids augmenting the already significant effects of diet. Two-way ANOVA analyses confirmed a significant positive interaction between diet and exercise in reducing TC and LDL and preventing plaque accumulation in the aortic arch. Triglyceride and VLDL levels were lowered by exercise with both diets but in this case there was no interaction suggesting independent roles for the interventions in regulating these lipids. Whereas we focused our studies on the aortic arch where the most dense plaque accumulates and is a strong predictor of adverse clinical events [for example see ref. 37], similar trends were seen for total plaque across the entire aorta (Fig. 7 and data not shown). A compounding influence on the potentially positive effects of exercise alone may be ad-libitum access to food, a condition designed to mimic human subjects that embark on exercise regimens without limiting caloric intake. We found that exercise significantly increased food intake of both diet groups although there was also a significant interactive effect, with mice in the HF-E group consuming a small but significantly greater amount of chow than the HP-E group. This may be related to taste and food preference by mice in the HF group. Despite increased food consumption, mice in both exercise groups demonstrated significantly lower weight gain (both about 10%) compared with sedentary mice. Most importantly, only mice that were simultaneously switched to the HP diet showed a significant benefit of exercise on plaque accumulation and this correlated with a similar positive interaction of diet and exercise on TC and LDL as well as the inflammatory markers IL-6 and CRP. Therefore, whereas energy expenditure more than offset the additional calories consumed, when the increased calories were from the HF diet they appear to neutralize the positive effects of exercise on lipids and atherosclerosis prevention. In this model, voluntary exercise lowered IL6 and CRP in both diet groups but did not reduce the progression of atherosclerosis in the absence of dietary intervention. We found a small but significant decrease in the level of HDL in the HP-E group. Whereas we do not know the reason for this, HDL levels have been reported to increase with exercise in human subjects (reviewed in [39]), but may be reduced by low fat diets [34], [35].

Voluntary exercise in mice with unrestricted access to the exercise wheel is equivalent to a strenuous aerobic exercise program. Mice run an average of 6 Km per day and spend approximately 4 h running per 24 h at a mean speed of 1.5 Km/hr (data not shown). Our results differ from other reports on the effects swimming exercise on ApoE-/- mice maintained on a continuous HF diet. The latter studies reported that atherosclerotic plaque accumulation was attenuated by 30-minute swimming periods 3 times per week for 8-weeks, with no change of lipids. The effects of exercise in this study were attributed to enhanced anti-oxidant and NO production [32], [33]. We also found that exercise did not reduce the levels of TC or LDL when mice were fed a continuous HF diet, in fact there was a trend for these to be increased, most likely caused by the significantly increased food intake associated with exercise. Also in our studies exercise only prevented plaque accumulation when mice were switched from HF to HP. The differences may involve the nature of the exercise; swimming periods were of shorter duration but may be more intense than voluntary running. The swim studies did not report food intake but they reported no change in average body weight associated with exercise suggesting that there are major differences in activity level and “lifestyles” between the swim protocol and our studies on voluntary exercise. Also in our studies the mice were older and heavier with 4.5-months of pre-formed plaque before exposure to diets and exercise. Plaque deposition involves multiple steps beginning with inflammation and loss of endothelial integrity, followed by lipid and inflammatory cell infiltration, deposition of fatty streaks and ultimately foam cell production and neointimal expansion (reviewed in [40]–[42]). We found that atherogenic lipid levels correlated more closely with plaque accumulation than did the IL6 or CRP levels in exercised mice fed HP suggesting that lipid regulation is more important than inflammation in regulating plaque progression in this model.

Our results are consistent with most of the studies on the effects of exercise on CAD patients but perhaps at variance with the concept that exercise programs alone are always protective against CAD [43]–[45]. The recently updated Cochrane Collaboration review analyzed the effectiveness of exercise-based cardiac rehabilitation in patients with coronary heart disease [46], [47]. From 48 trails and 8940 subjects it was found that long-term exercise programs significantly reduced cardiac mortality as well as mean cholesterol and triglyceride levels (−14.3 mg/dL and −20.4 mg/dL respectively), but no significant changes of HDL or LDL. Significantly lower rates of self-reported smoking further suggest the presence of subgroups with self-imposed lifestyle modifications that may include changed eating habits and diet. These results are also consistent with the analyses of Leon & Sanchez [19] where less than 50% of 2200 subjects recruited to exercise training programs without diet modification displayed significant reductions of TG, LDL or TC. It seems possible that within these groups there are subjects with severe dyslipidemia and advanced CAD that are unresponsive to even intensive long-term aerobic training without concurrent diet and lifestyle modifications.

In conclusion, there is increasing evidence that exercise training can reduce endothelial dysfunction and the progression of atherosclerosis. Exercise training improves the bioavailability of nitric oxide, diminishes the level of inflammatory markers, and can enhance the numbers of circulating endothelial progenitor cells [48] while reducing EPCs in the bone marrow [38], with the potential of reducing atherosclerosis [49]. Results presented here suggest that in atherosclerosis-prone ApoE-/- mice the protective effects of aerobic exercise over an extended period may be significantly diminished by diet and pre-elevated levels of atherogenic lipids. We only observed significant TC/LDL-lowering and decreased atherosclerosis by exercise when the exercise program was complemented by a heart-healthy diet enriched in protein and unsaturated oils. Despite significantly lower body weights and inflammation markers in exercised mice on the high fat diet, plaque accumulation was not significantly reduced. Therefore it seems possible that in this model where significant disease is already present, protection against further plaque accumulation and plaque regression requires correction of lipid and cytokine profiles, conditions seen only when exercise was superimposed on the high protein diet.
